# Pediatric lung adenocarcinoma presenting with brain metastasis: a case report

**DOI:** 10.1186/s13256-018-1781-1

**Published:** 2018-09-02

**Authors:** Lucia De Martino, Maria Elena Errico, Serena Ruotolo, Daniele Cascone, Stefano Chiaravalli, Paola Collini, Andrea Ferrari, Paolo Muto, Giuseppe Cinalli, Lucia Quaglietta

**Affiliations:** 1 Department of Pediatric Oncology, Santobono-Pausilipon Children’s Hospital, Posillipo Street, 226, 80122 Naples, Italy; 2 Department of Pathology, Santobono-Pausilipon Children’s Hospital, Naples, Italy; 3 Department of Neuroscience, Santobono-Pausilipon Children’s Hospital, Naples, Italy; 4 Department of Pediatric Oncology, Fond. IRCCS Istituto Nazionale dei Tumori, Milan, Italy; 5 Soft Tissue and Bone Pathology, Histopathology and Pediatric Pathology Unit, IRCCS Istituto Nazionale Tumori, Milan, Italy; 6 Radiation Oncology Unit, National Tumor Institute of Naples, Foundation G. Pascale, Naples, Italy; 7 Department of Pediatric Neurosurgery, Santobono-Pausilipon Children’s Hospital, Naples, Italy

**Keywords:** Brain metastasis, Cancer immunotherapy, Lung adenocarcinoma, Nivolumab, Rare tumor, TREP

## Abstract

**Background:**

Diagnosis and treatment of primary lung adenocarcinoma in children remains challenging given its rarity. Here we highlight the clinical history, pathological evaluation, genomic findings, and management of a very young patient with metastatic lung adenocarcinoma.

**Case presentation:**

A 10-year-old white girl presented with brain metastases due to primary pulmonary adenocarcinoma. Next generation sequencing analysis with “Comprehensive Cancer Panel” highlighted the presence of multiple non-targetable mutations in the *FLT4*, *UBR5*, *ATM*, *TAF1*, and *GUCY1A2* genes. She was treated aggressively with chemotherapy, surgery, and radiation therapy for local and distant recurrence. Eventually, therapy with nivolumab was started compassionately, and she died 23 months after diagnosis.

**Conclusions:**

Extremely rare cancers in children such as lung adenocarcinoma need accurate and specific diagnosis in order to develop an optimal plan of treatment. It is also necessary to underline that “children are not little adults,” thus implying that an adult-type cancer in the pediatric population might have a different etiopathogenesis. Diagnostic confirmation and primary treatment of such rare conditions should be centralized in reference centers, collaborative networks, or both, with multidisciplinary approaches and very specific expertise.

## Background

Primary lung neoplasms are extremely rare in the pediatric population with an overall incidence of less than 0.05 per 100,000 cases [1–4]. According to the World Health Organization (WHO) classification, malignant pulmonary tumors of epithelial origin include non-small cell lung cancer (NSCLC), further subdivided into adenocarcinoma, squamous cell carcinoma, and large-cell carcinoma; other carcinomas of the lung are small cell lung cancer (SCLC) or neuroendocrine tumors [5]. In childhood, pulmonary adenocarcinoma constitutes approximately 15% of all pulmonary malignant tumors [3]. The incidence of brain metastases is very low in children with solid primary tumors and has previously been estimated to be 1.5 per 100,000 in children aged 0–14 years at diagnosis [6]. However, the United States Cancer Registry reported that between 1973 and 2011, brain metastases from NSCLC developed in approximately 9% of all pediatric cases [7]. To date, only a few case reports and small case series have been published, and, as such, the treatment choice for lung adenocarcinoma in children is extrapolated from studies in adult patients [1, 2, 4, 8–17]. Here we describe a 10-year-old white girl who presented with brain metastasis from primary pulmonary adenocarcinoma and was treated aggressively, including radiation therapy, for local and distant recurrence and programmed cell death-1 (PD-1) inhibitor therapy. She died 23 months after diagnosis.

## Case presentation

**Fig. 1:**
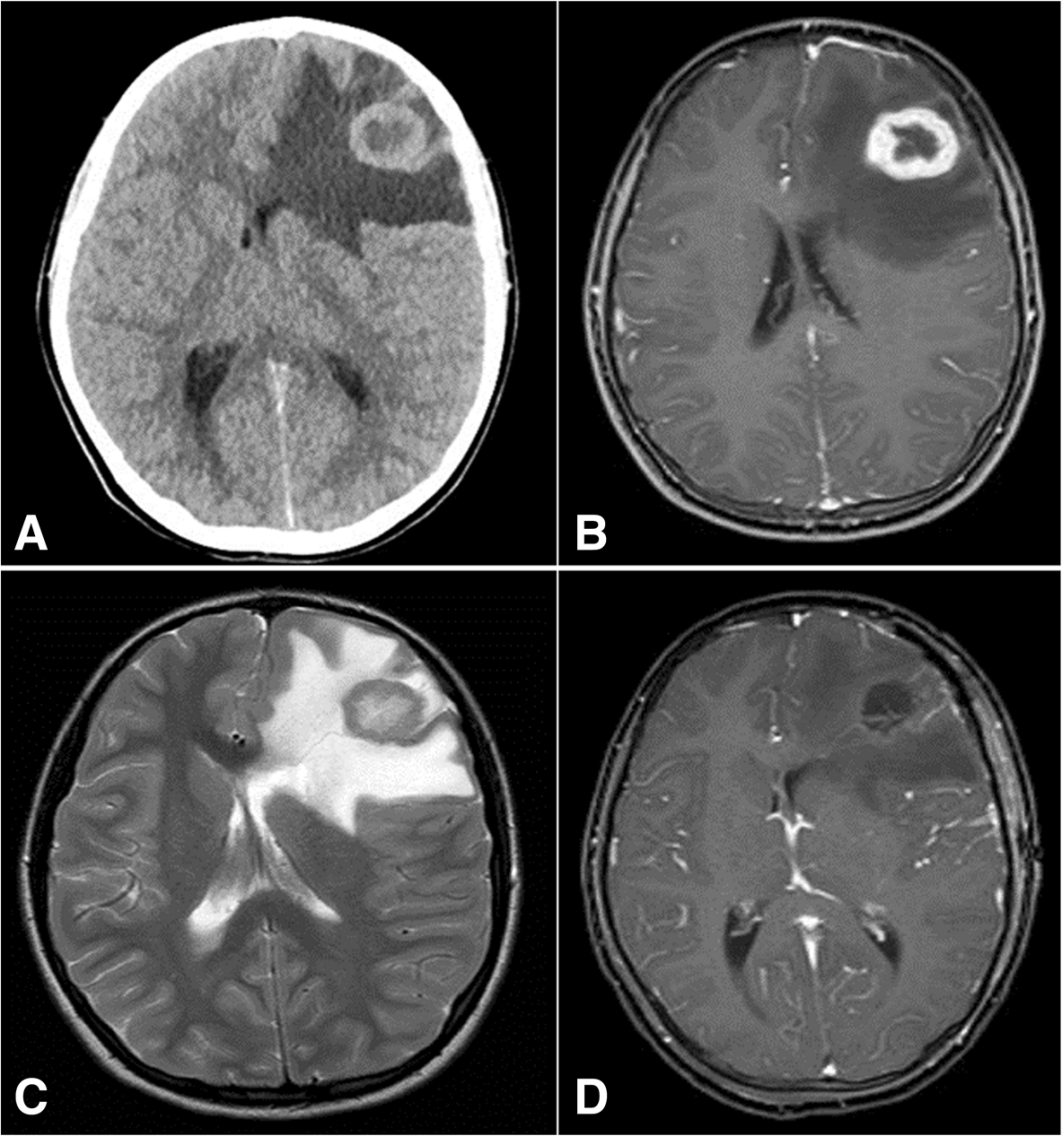
Brain metastasis of lung adenocarcinoma at diagnosis on non-enhanced computed tomography scan (**a**), post-contrast T1-weighted (**b**), fluid-attenuated inversion recovery magnetic resonance imaging (**c**) and after surgical resection on post-contrast T1-weighted (**d**)

A 10-year-old white girl presented to our emergency room in January 2015 with a 1-month history of headache and morning vomiting. On examination, she appeared slightly pale, with body temperature of 36.5 °C, heart rate of 90 beats per minute, blood pressure of 106/62 mmHg, respiratory rate of 18 breaths per minute, and oxygen saturation of 100% in ambient air. Her neurological status was normal. Laboratory test results are shown in Table 1. A chest X-ray was within limits. An urgent non-enhanced brain computed tomography (CT) scan showed a focal lesion in the left frontal subcortical region with prominent surrounding edema and mass effect (Fig. 1a). She was therefore admitted to our hospital. Magnetic resonance imaging (MRI) demonstrated ring enhancement on post-contrast T1-weighted (T1W) sequences; fluid-attenuated inversion recovery (FLAIR) sequences confirmed extensive vasogenic edema (Fig. 1b-c). She lived with her parents and siblings in Southern Italy. Before the onset of the current illness, at 5 years of age she had undergone surgical excision of a pleomorphic adenoma of the parotid gland. No evidence of a pre-existing congenital airway malformation was referred. She was not sexually active, and she did not smoke cigarettes, drink alcohol, or use illicit drugs. Her father, a heavy tobacco smoker, was a merchant. Her mother, a housewife, reported three miscarriages. Her maternal grandfather had died from colon cancer at 40 years. Her paternal aunt was affected by Hodgkin lymphoma, and a second-degree cousin presented ovarian immature teratoma. After multidisciplinary discussion, neuronavigation and left frontal craniotomy with tumor resection with direct cortical and subcortical stimulation was done under general anesthesia. She received preoperative steroid medication which was tapered post-surgery. MRI scanning within 72 hours after surgery documented total resection (Fig. 1d).

**Table 1:** Laboratory data on presentation

Variable	Values
Hemoglobin (g/dl)	13.9
Hematocrit (%)	38.4
White cell count (per mm^3^)	12,530
Differential count (%)	
- Neutrophils	74.1
- Lymphocytes	21
Platelet count (per mm^3^)	319,000
Red cell count (per mm^3^)	4,960,000
Mean corpuscular volume (fl)	77.4
Sodium (mmol/liter)	140
Potassium (mmol/liter)	4.2
Chloride (mmol/liter)	101
Calcium (mg/dl)	10
Phosphorus (mg/dl)	4.6
Glucose (mg/dl)	83
Urea nitrogen (mg/dl)	32
Creatinine (mg/dl)	0.67
Protein total (g/dl)	7.6
Albumin	4.7
Alanine aminotransferase (U/liter)	20
Aspartate aminotransferase (U/liter)	20
Bilirubin total (mg/dl)	0.56
Lactate dehydrogenase (U/liter)	488
Creatine kinase (U/liter)	77
C-reactive protein (mg/liter)	0.17
Urine analysis	Normal
Serology for toxoplasmosis, herpes virus, CMV, EBV, rubella	Negative

*CMV* cytomegalovirus, *EBV* Epstein–Barr virus

**Fig. 2:**
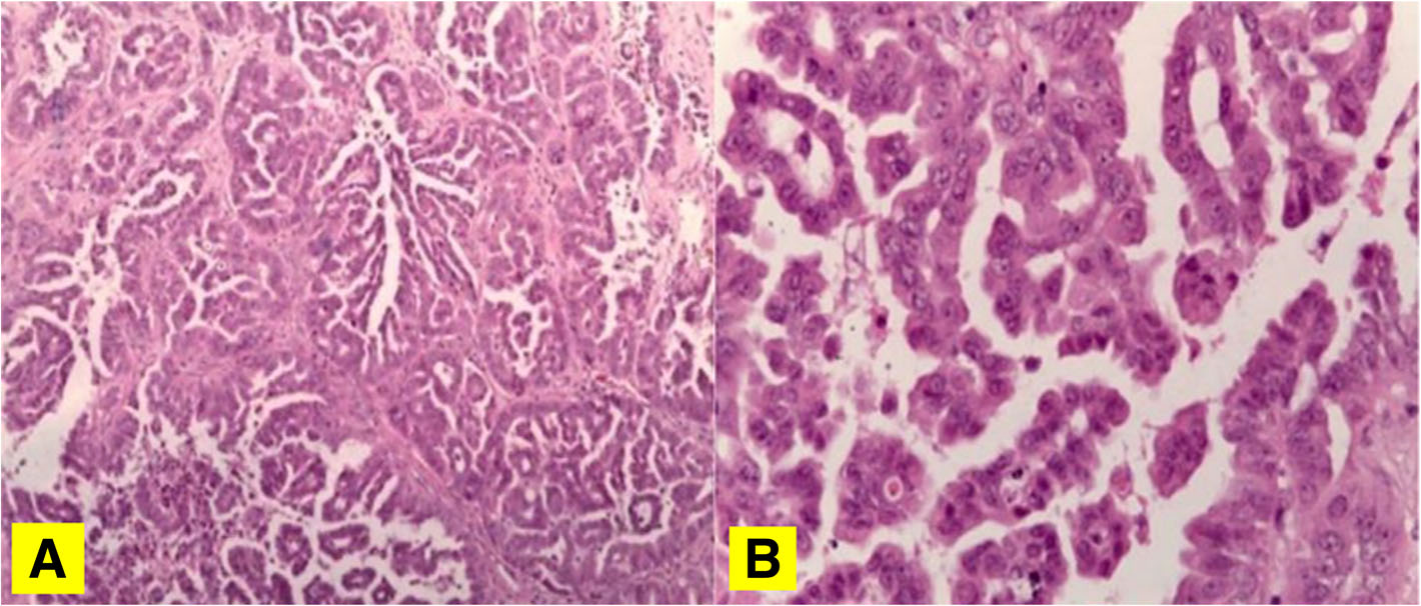
Lung adenocarcinoma with micropapillary pattern (hematoxylin and eosin, × 10) (**a**) and cytological details (hematoxylin and eosin, × 40) (**b**)

**Fig. 3:**
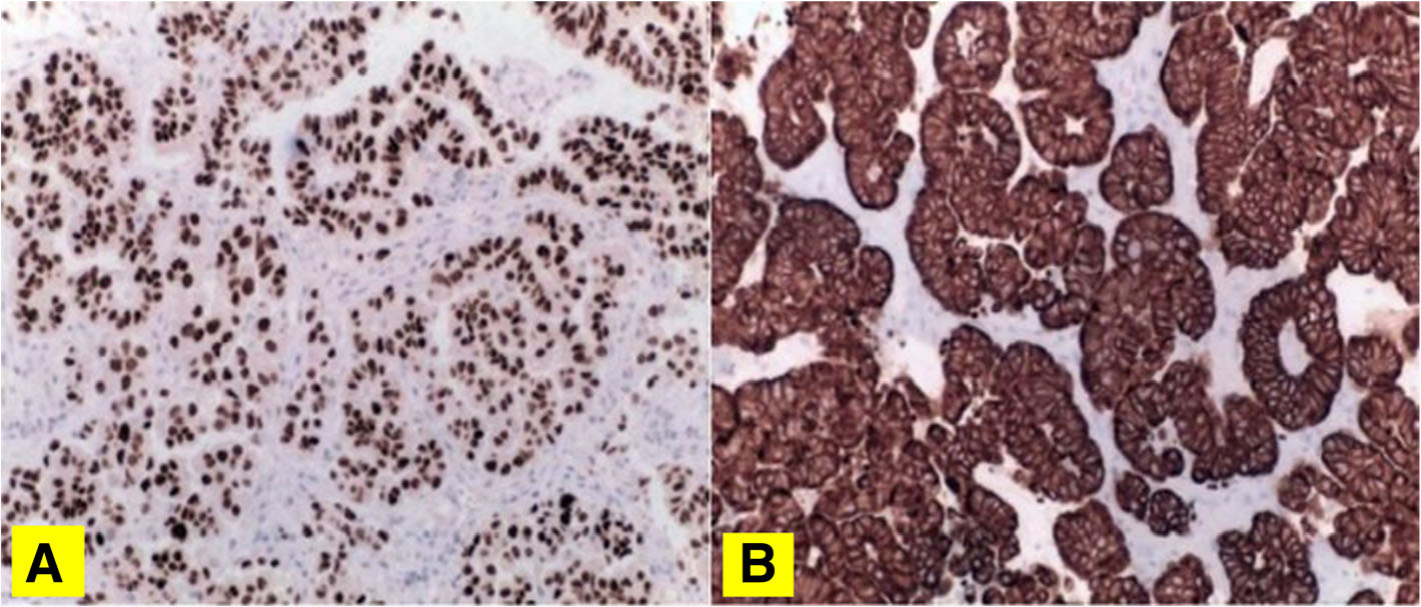
Immunoreactivity for thyroid transcription factor 1 (**a**) and cytokeratin 7 (× 400) (**b**)

**Fig. 4:**
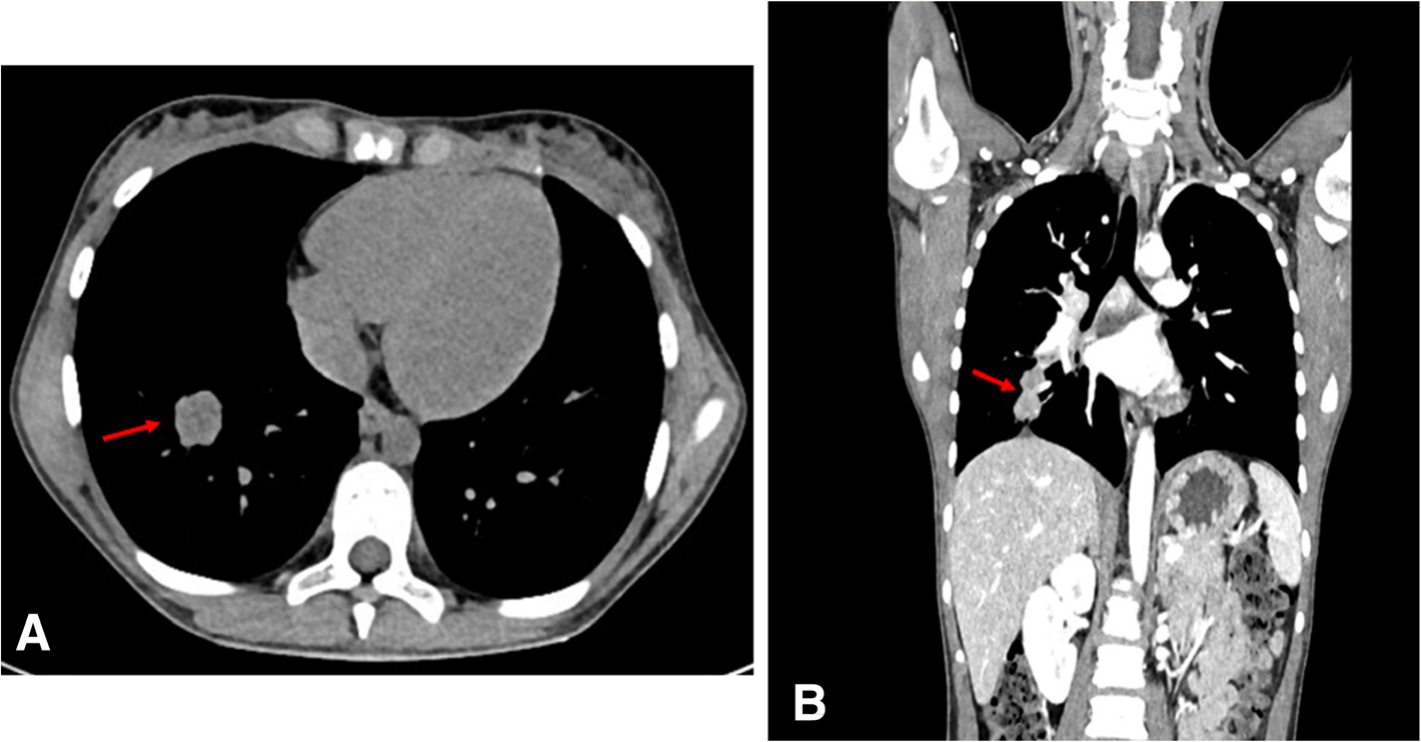
Lung adenocarcinoma in the lower lateral basal segment of the right lobe (red arrow) in our 10-year-old white girl on axial (**a**) and coronal (**b**) computed tomography scan

**Fig. 5:**
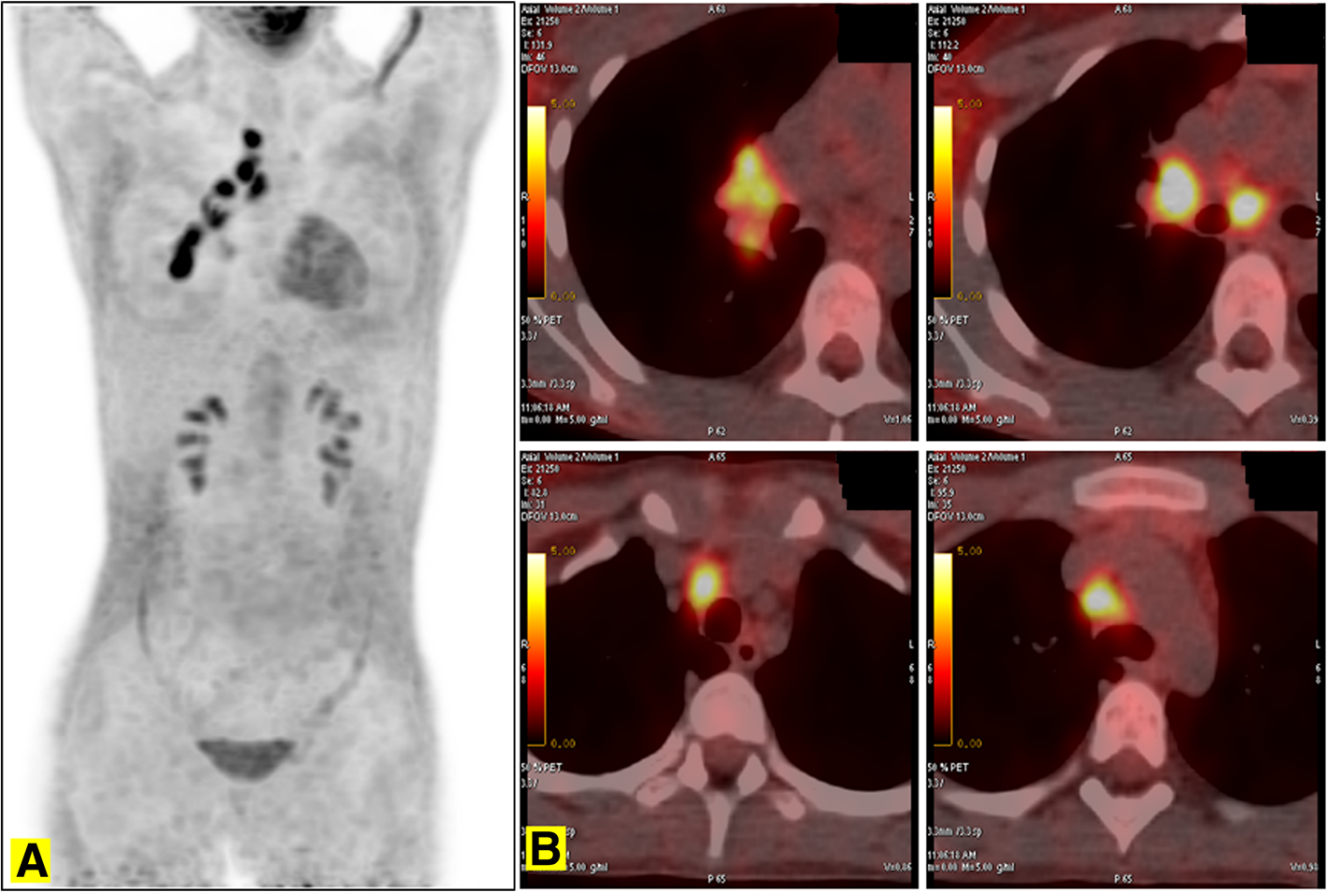
Thoracic positron emission tomography scan (**a**) and positron emission tomography/computed tomography scans (**b**) performed for the staging of lung adenocarcinoma

Microscopy on tissue sections showed malignant neoplasms with extensive necrosis, composed of atypical columnar and cuboidal cells, which had vesicular nucleolated nuclei and eosinophilic cytoplasm. Tumor cells covered papillary structures with fibrovascular cores or formed small glands and micropapillae lacking stroma. The surrounding brain parenchyma showed evidence of reactive gliosis and lymphohistiocytic infiltrate (Fig. 2). On immunohistochemical examination, neoplastic cells were positive for cytokeratin 7, thyroid transcription factor 1 (TTF-1) (Fig. 3), cytokeratin AE1/AE3, and epithelial membrane antigen **(**EMA), whereas all other markers tested were negative: cytokeratin 20, carcinoembryonic antigen **(**CEA), thyroglobulin, vimentin, cluster of differentiation **(**CD) 10, WT1, calretinin, inhibin, CD117, CD30, S100 protein, melan-A, actin, chromogranin, synaptophysin, and glial fibrillary acidic protein **(**GFAP). INI1 expression was retained. Thus, a diagnosis of metastatic lung adenocarcinoma was proposed. A chest CT scan showed a parenchymal nodular lesion in the lower lateral basal segment of the right lobe, measuring 32 mm × 18 mm × 17 mm, thought to be the primary lung cancer (Fig. 4) with mediastinal nodal metastasis. Tumor spread was confirmed by positron emission tomography (PET)/CT showing a primary lung tumor and mediastinal lymph nodes with high fluorodeoxyglucose (FDG) uptake: maximum standardized uptake value (SUVmax) of 8.5 and 8, respectively (Fig. 5).

At fluorescence *in situ* hybridization (FISH) analysis, no rearrangements of anaplastic lymphoma kinase (*ALK*), c-ros oncogene 1, receptor tyrosine kinase (*ROS1*), and rearranged during transfection (*RET*) genes were found. *ROS1* gene was found deleted in 57% of neoplastic cells. Next generation sequencing (NGS) analysis was applied to genomic deoxyribonucleic acid (DNA) extracted from formalin-fixed paraffin-embedded tissue. Both the “Cancer Hotspot Panel” (50 genes) and the “Comprehensive Cancer Panel” (444 genes) through the Personal Genome Machine with Ion Torrent™ technology (Life Technologies, Applied Biosystems) were applied. NGS analyses with Comprehensive Cancer Panel highlighted the presence of multiple non-targetable mutations in fms-related tyrosine kinase 4 (*FLT4*), ubiquitin-protein ligase E3 component N-recognin 5 (*UBR5*), ataxia telangiectasia mutated (*ATM*), and TATA-box binding protein associated factor 1 (*TAF1*). Epidermal growth factor receptor (*EGFR*) mutation status was negative.

**Fig. 6:**
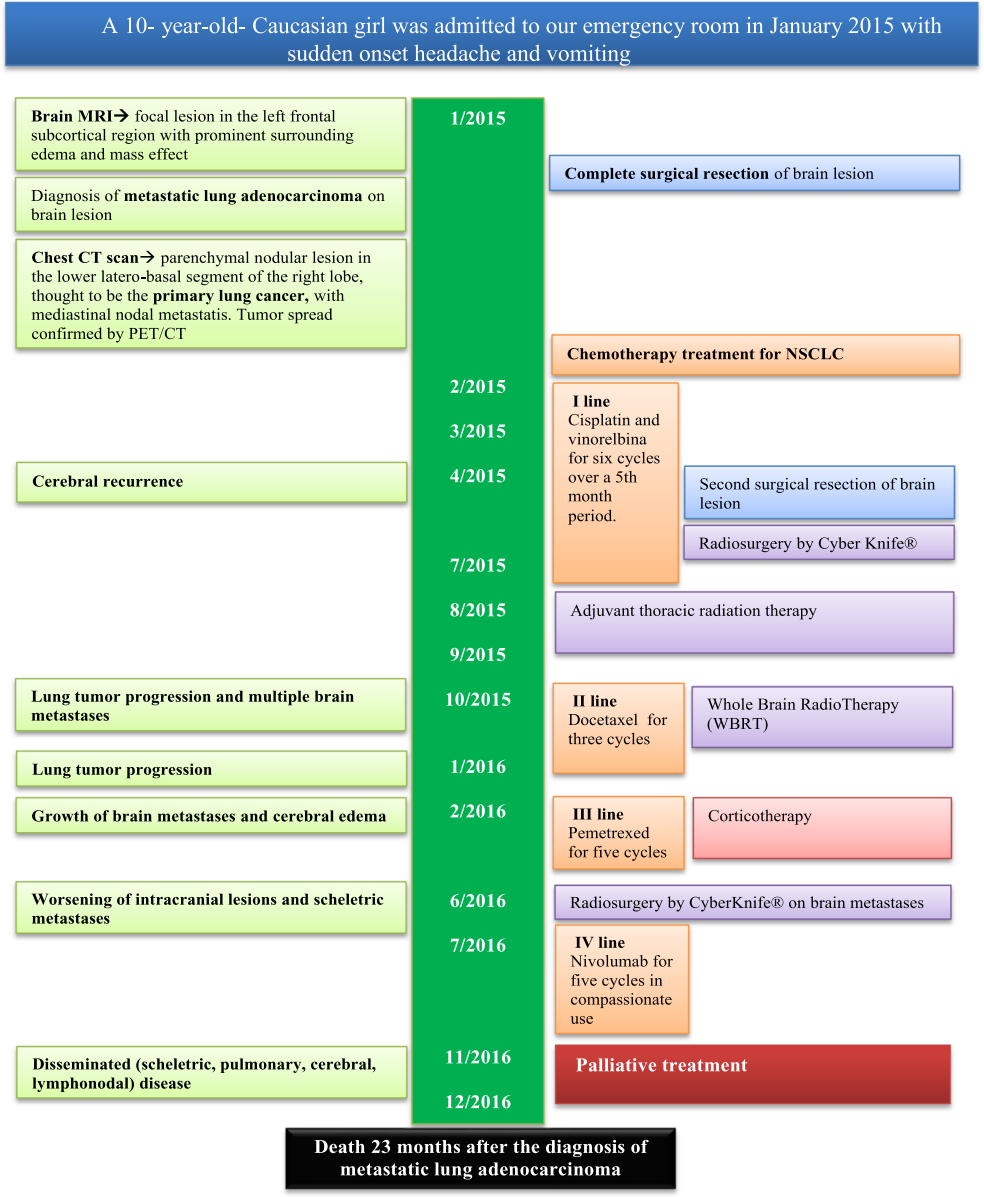
Medical history timeline. *CT* computed tomography, *MRI* magnetic resonance imaging, *NSCLC* non-small cell lung cancer, *PET* positron emission tomography

One month after admission our patient started chemotherapy treatment for NSCLC with cisplatin and vinorelbine for six cycles over a 5-month period. Two months later, an MRI 3 months after diagnosis revealed cerebral recurrence; therefore, she underwent a second surgical resection, followed by radiosurgery (CyberKnife). A brain MRI and PET/CT scan after completion of her last dose of chemotherapy showed absence of cerebral metastasis and partial regression of the lesion of the lower lobe of her right lung (RLL); thus, between 7 and 8 months after admission she received adjuvant thoracic radiation therapy. Unfortunately, 1 month later surveillance imaging revealed lung tumor progression and multiple brain metastases. She subsequently started whole brain radiotherapy (WBRT) and three cycles of docetaxel. One year after admission a rapid lung tumor progression was documented. One month later she developed headache and vomiting due to increased cerebral edema and growth of brain metastases. Therefore, she started corticotherapy and third-line pemetrexed treatment (five cycles), but 5 months later a PET/CT scan revealed further worsening of intracranial lesions and skeletal metastases. She underwent radiosurgery by CyberKnife technique on brain metastases and the following month she received nivolumab at 3 mg/kg intravenously every 2 weeks compassionately. Due to worsening of clinical conditions, a month later PET/CT was performed, revealing disseminated (skeletal, pulmonary, cerebral, lymphonodal) disease. She continued nivolumab, receiving a total of five cycles without adverse events. Given the ongoing clinical and imaging deterioration, palliative treatment was initiated and she died of respiratory failure 23 months after diagnosis of metastatic lung adenocarcinoma (Fig. 6). Autopsy was declined by parents.

## Discussion

A 10-year-old white girl presented to our hospital with brain metastases from primary pulmonary adenocarcinoma. She was treated aggressively with chemotherapy, neurosurgery, and radiation therapy for local and distant recurrence. At the end, therapy with nivolumab was started for compassionate use. However, she died 23 months after diagnosis. To the best of our knowledge, this child would be the youngest patient with primary adenocarcinoma of the lung presenting with brain metastasis so far described.

The diagnosis and treatment of primary lung adenocarcinoma in children is challenging due to the rarity of the disease. The Surveillance, Epidemiology, and End Results (SEER) program of the National Cancer Institute (NCI) collected data on 40 patients (< 15-years old) who were diagnosed from 1984 to 2014 as having NSCLC of which 22 (55%) patients were affected by mucoepidermoid carcinoma and 18 (45%) by adenocarcinoma. In this group, 44% (8/18) of patients presented distant metastasis. There is evidence that genetic factors may play a role in the development of lung adenocarcinoma in young patients who have never smoked tobacco, and familial genetic clustering of lung cancer has been found [18, 19]. In our patient, somatic non-targetable mutations in *FLT4*, *UBR5*, *ATM*, *TAF1*, and *GUCY1A2* genes were identified. In particular, *ATM* has a key role in maintaining genomic integrity and it is hypothesized that early loss of *ATM* function in the process of malignant transformation could be responsible for the generation of a mutator phenotype, an “enabling feature” in the growth of cancer [20]. Recently, an *ATM* germline polymorphism has been significantly associated with lung cancer susceptibility [21]. However, no germline mutations have been studied in this child.

Primary lung carcinoma in children usually has an aggressive growth pattern with high mortality (90%), and average survival of 7 months after diagnosis [1, 2]. Primary lung adenocarcinoma in children has the poorest prognosis with a 5-year overall survival of approximately 25% [3]. Due to the rarity of this condition in children, no clinical trials have been performed: almost an orphan disease, treatment strategies are extrapolated from studies conducted in adult populations. For adult cancers, nowadays targeting the immune system to control tumor growth and metastasis is intuitively appealing, as this represents supporting body defenses instead of the usual weakening that is brought about by toxic conventional therapy. In this context, new therapies have emerged recently, among which are PD-1 immune checkpoint inhibitors, such as nivolumab [22]. In particular, nivolumab monotherapy has been registered as a second-line therapy for patients with NSCLC of squamous cell histology, unrestricted for programmed cell death-ligand 1 (PD-L1) status, and nonsquamous histological subtypes selected for PD-L1 status (> 1%) [22]. In considering this promising therapeutic approach, a key issue is whether biomarkers recommended in adult cancers are present in childhood tumors as well. In fact, the impact of targeted agents in the pediatric population has not paralleled the progress seen in adult patients. Moreover, children differ from adults in many aspects of pharmacotherapy, including capabilities for drug administration, medicine-related toxicity, and taste preferences. New law regulations and collaborative research between pediatric oncologists and adult experts should be reinforced and supported in order to develop pediatric-targeted therapies, especially for adult-type cancers occurring in children.

This case report has several limitations. First, we established a diagnosis of lung adenocarcinoma on the brain metastasis and we did not perform a biopsy of the primary lesion or recurrences. A biopsy of the primitive tumor could have given more information about the histology of lung cancer, for example a combined histology that was not apparent on the metastatic biopsy sample. Moreover, whereas biopsy at progression in NSCLC is not generally indicated, since the tumor in our patient did not respond as initially expected, a repeat biopsy might have ruled out a shift from NSCLC to SCLC and could have assessed targeted pathways not present on the first biopsy. Second, we applied NGS to the cancer sample to identify eventually targeted therapy; however, the extension of NGS analysis to a non-tumoral sample, although not useful in terms of treatment, could have detected lung cancer susceptibility; this information would have been useful in order to improve clinical management of our patient and her family and to enhance knowledge in the field of rare tumors in the pediatric population. Third, as PD-1/PD-L1 inhibitors have been rapidly integrated into standard of care for NSCLC, we used nivolumab in our patient in compassionate use; however, we did not assess PD-L1 status because we decided to use it irrespectively of its result.

## Conclusions

The present case emphasizes the need to consider unusual presentations of rare cancers in the differential diagnosis of more common diseases in pediatric patients, especially because early diagnosis and active treatment are vital to improve prognosis and survival. Notably, it is mandatory to establish promptly an accurate and specific diagnosis to develop an optimal plan of treatment and, to the extent possible, estimate prognosis. Genomic sequencing technologies applied to tissue specimens are able to detect targetable driver mutations and the application of these analytical approaches to non-tumoral samples could allow the identification of germline mutations that predispose individuals to lung cancer [21]. Moreover, biopsy of the primitive tumor and progression should be considered. In fact, treatment strategies for lung cancer are based on the hypothesis that an individual patient’s cancer is purely of one subtype. However, because of the clinical history (presence of cancer in familial history), clinical characteristics (very young age, no signs of genetic syndromes), and the partial response to chemotherapy, a different etiopathogenesis should be considered in our patient. In fact, recent evidence shows that NSCLC and SCLC, although considered different entities with distinct biology and genomic abnormalities, might share common cells of origin and they might present with a combined histology and/or tumor transformation from a subtype to another [23]. Finally, diagnostic pathological confirmation and primary treatment of rare cancers in children, in particular, should be centralized to reference centers, collaborative networks, or both, with multidisciplinary approaches and very specific expertise. In this regard, a national cooperative project on rare pediatric tumors (the TREP project) was launched in 2000 in Italy, with a view to improving the clinical management and the basic research on these “orphan” tumors; “orphan” tumors are defined as those childhood solid malignancies characterized by an annual incidence ≤2/million inhabitants and not considered in other clinical trials [24]. Moreover, national groups working in Italy, France, UK, Poland, and Germany should join forces in the European Cooperative Study Group for Pediatric Rare Tumors (EXPeRT), with the ultimate goal that children with very rare tumors may benefit from a closer knit and stronger international network, thanks also to the fact that the project is now officially supported by the International Society of Paediatric Oncology [25].

## Abbreviations

CD: Cluster of differentiation
CT: Computed tomography
MRI: Magnetic resonance imaging
NGS: Next generation sequencing
NSCLC: Non-small cell lung cancer
PD-1: Programmed cell death-1
PD-L1: Programmed cell death-ligand 1
PET: Positron emission tomography
SCLC: Small cell lung cancer
